# circARID1A Inhibits Tail Fat Cell Differentiation in Guangling Large-Tailed Sheep by Regulating the miR-493-3p/YTHDF2 Axis

**DOI:** 10.3390/ijms252212351

**Published:** 2024-11-18

**Authors:** Yan Shen, Yu Liang, Zikun Yuan, Liying Qiao, Jianhua Liu, Yangyang Pan, Kaijie Yang, Wenzhong Liu

**Affiliations:** College of Animal Science, Shanxi Agricultural University, Jinzhong 030801, China

**Keywords:** Guangling Large-Tailed sheep, adipocyte differentiation, circARID1A, miR-493-3p, YTHDF2

## Abstract

The Guangling Large-Tailed sheep is renowned for its unique tail fat deposition, with a significant proportion of its total body fat being localized in the tail region. Fat deposition is a complex biological process regulated by various molecular mechanisms. Our previous studies have identified a large number of differentially expressed circular RNAs (circRNAs) in the tail adipose tissue of the Guangling Large-Tailed sheep. These circRNAs may play a pivotal role in the process of fat deposition. Given the potential regulatory functions of circRNAs in adipose metabolism, investigating their roles in tail fat deposition is of significant scientific importance. In this study, we identified novel circARID1A. Using various experimental methods, including lentivirus infection, RNase R treatment, actinomycin D assay, qPCR, western blotting, and dual-luciferase reporter assays, we determined that circARID1A inhibits the expression of miR-493-3p through competitive binding, thereby regulating adipocyte differentiation. Further research revealed that miR-493-3p promotes adipocyte differentiation by targeting YTH domain family 2 (YTHDF2), and this regulatory effect is also influenced by circARID1A. In conclusion, our findings suggest that circARID1A inhibits tail fat cell differentiation in the Guangling Large-Tailed sheep through the circARID1A/miR-493-3p/YTHDF2 axis, providing theoretical support for improving meat quality and fat deposition in sheep.

## 1. Introduction

The sheep industry is a vital part of animal husbandry, providing meat, milk, wool, and other animal products. Through natural selection and artificial breeding over time, different sheep breeds have developed distinct body shapes and physical characteristics, resulting in various tail types [1]. The Guangling Large-Tailed sheep is a breed characterized by its fatty tail. Its slaughter performance results from the interplay of multiple factors, including the degree of muscle development, the uniformity of fat distribution, and the moisture and protein content in the meat. These factors not only directly affect the texture and nutritional value of the meat but also influence slaughter yield and the proportion of edible portions, which are critical for economic benefits. Research indicates that fat deposition in sheep occurs in a specific sequence, with tail fat accumulating first and more efficiently than in other tissues [2]. Therefore, as a typical fatty-tail breed, studying the molecular mechanisms of tail fat deposition in the Guangling Large-Tailed sheep can provide a more detailed theoretical basis for optimizing meat quality and enhancing production efficiency. Currently, research on the molecular mechanisms of tail fat deposition in this breed is relatively limited, particularly regarding the gene regulatory networks involved in adipogenesis, which remain insufficiently explored. A deeper understanding of these mechanisms will not only aid in improving meat quality and reducing excessive fat deposition but also provide more precise molecular markers for breeding, thus enhancing production efficiency and lowering breeding costs. Therefore, uncovering the molecular mechanisms associated with tail fat accumulation is of significant practical importance for improving the production efficiency of the Guangling Large-Tailed sheep.

Fat deposition is a biological phenomenon regulated by complex processes, among which non-coding RNAs (ncRNAs) play a crucial role in adipocyte differentiation [3]. Circular RNAs (circRNAs) are endogenous, covalently closed-loop ncRNAs that can act as “sponges” for microRNAs (miRNAs) in eukaryotic cells, thereby enhancing the expression of target genes. Molecular sponges function by adsorbing specific miRNA molecules, thereby preventing miRNAs from binding to the 3′ untranslated region (3′UTR) of their target genes. Unlike linear RNAs, circRNAs form closed loops through covalent bonding of their 3′ and 5′ ends, making them more stable [4]. The first covalently closed circRNA was discovered in a plant virus in 1976 [5]. Early studies suggested that circRNAs lacked function, but further research has revealed that circRNAs perform various essential functions in biology through multiple pathways [6]. Recently, the role of circRNAs as competing endogenous RNAs (ceRNAs) and their impact as miRNA sponges on target gene expression has garnered significant attention [7]. For example, studies have shown that circPPARA can absorb miR-429 and miR-200, promoting intramuscular fat production in pigs [8]; sus_circPAPPA2 acts as a molecular sponge for miR-2366, enhancing the expression of glucose kinase (GK) and promoting preadipocyte differentiation in pigs [9]; circFUT10, as a molecular sponge for let-7, promotes adipocyte proliferation and inhibits adipocyte differentiation in cattle [10]; and circRNA_2837 binds to miR-34a, reducing its inhibition of genes related to adipocyte differentiation, thus inhibiting adipocyte differentiation [11]. These studies suggest that circRNAs play a significant role in fat generation through the circRNA–miRNA–mRNA regulatory network. However, research on the role of circRNAs in tail fat deposition in the Guangling Large-Tailed sheep is still limited.

Our research group identified several circRNAs through whole-transcriptome sequencing, among which circ_0002643 is located on the AT-interacting domain-rich protein 1A (*ARID1A*) gene and named circARID1A. Studies have shown that the loss of ARID1A directly affects the expression of target genes in adipogenesis and fatty acid oxidation pathways, leading to fatty liver disease [12]. Recent research indicates that miR-493-3p regulates multiple target genes in sheep skeletal muscle development through an miRNA-mediated ceRNA network [13]. Additionally, miR-493-3p has been identified as a direct upstream factor of YTHDF2, capable of inhibiting the proliferation and migration of prostate cancer cells. Specifically, miR-493-3p enhances m6A levels by downregulating YTHDF2, significantly inhibiting prostate cancer cell proliferation and migration [14]. Therefore, we hypothesize that circARID1A may act as a ceRNA in the miR-493-3p/YTHDF2 pathway, playing a role in adipogenesis.

This study reveals the potential regulatory relationship between circARID1A, miR-493-3p, and their target YTHDF2. Specifically, circARID1A acts as a “sponge” for miR-493-3p, upregulating the expression of YTHDF2, thereby inhibiting the differentiation of fat cells in the Guangling Large-Tailed sheep. The findings further elucidate the role of the circARID1A/miR-493-3p/YTHDF2 signaling axis in adipogenesis, deepening the understanding of circRNA’s regulatory role in fat synthesis. This provides an important theoretical basis for studying the molecular mechanisms of tail fat deposition in the Guangling Large-Tailed sheep.

## 2. Results

### 2.1. Characteristics of circARID1A

**Figure 1:**
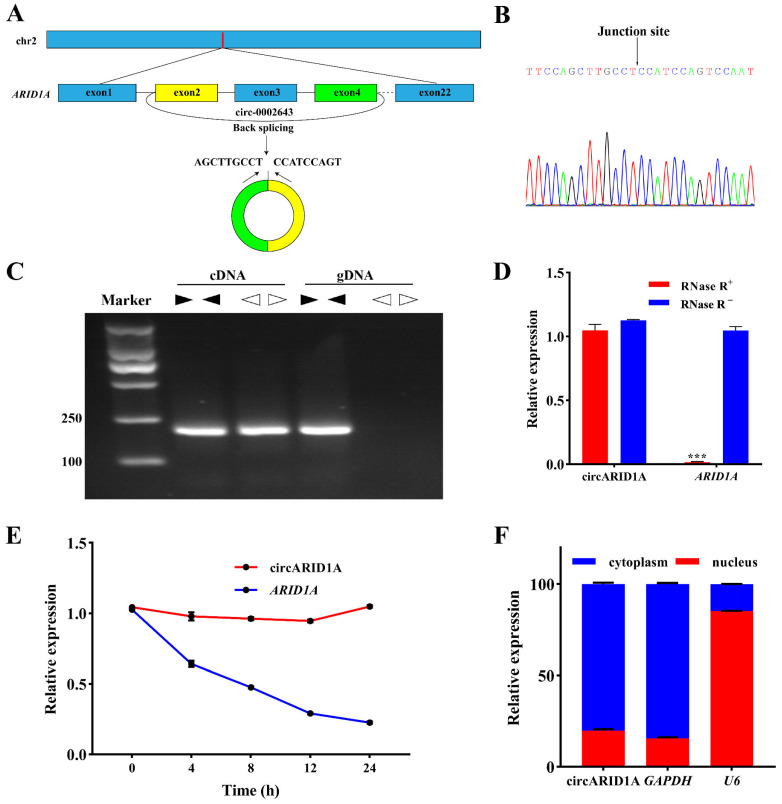
Characterization and identification of circARID1A. (**A**) circARID1A is generated from exons 2 and 4 of the *ARID1A* gene. (**B**) Sanger sequencing confirmed the presence of circARID1A. (**C**) PCR analysis using divergent and convergent primers on cDNA and gDNA. (**D**) Relative expression levels of circARID1A and ARID1A after RNase R treatment. (**E**) Relative expression levels of circARID1A and ARID1A after ActD treatment. (**F**) Relative expression levels of circARID1A in the nucleus and cytoplasm after nuclear–cytoplasmic separation. ***: *p* < 0.001.

Figure 1A illustrates the circularization process of circARID1A, and Sanger sequencing (Figure 1B) confirmed the presence of back-splicing products of circARID1A. Using divergent and convergent primers, PCR amplification was performed on genomic DNA (gDNA) and complementary DNA (cDNA) templates. Agarose gel electrophoresis results showed that the amplification product of circARID1A primers was present only in cDNA and not in genomic gDNA (Figure 1C). RNA extracted and treated with RNase R revealed that circARID1A exhibited stronger resistance to digestion compared to linear ARID1A (Figure 1D). Furthermore, circARID1A demonstrated greater stability compared to ARID1A when treated with actinomycin D (ActD) (Figure 1E). In nuclear–cytoplasmic separation experiments, with Glyceraldehyde-3-phosphate dehydrogenase (*GAPDH*) and *U6* as reference genes, results indicated that ARID1A was primarily expressed in the nucleus, while circARID1A was predominantly expressed in the cytoplasm (Figure 1F).

### 2.2. Regulation of Adipocyte Differentiation by circARID1A

**Figure 2:**
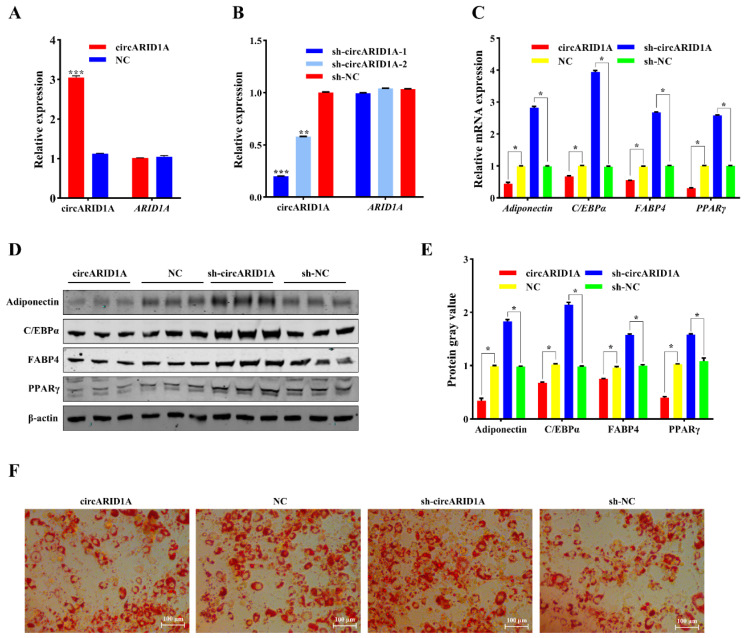
The effect of circARID1A on adipocyte differentiation. (**A**) Expression levels of circARID1A and ARID1A mRNA in adipocytes after transfection with circARID1A overexpression vector and control vector NC. (**B**) Expression levels of circARID1A and ARID1A mRNA after transfection with shRNA and sh-NC. (**C**) mRNA expression levels of four adipogenic marker genes after circARID1A overexpression and knockdown. (**D**,**E**) Protein expression levels of four adipogenic marker genes after circARID1A overexpression and knockdown. (**F**) Oil Red O staining results of adipocytes differentiated for 6 days. *: *p* < 0.05, **: *p* < 0.01, ***: *p* < 0.001.

To investigate the role of circARID1A in adipocyte differentiation, we constructed an overexpression vector and two interference vectors. The overexpression vector (pCD2.1-circARID1A) and interference vectors (sh-circARID1A-1 and sh-circARID1A-2) were transfected into adipocytes. circARID1A expression significantly increased (*p* < 0.001) (Figure 2A) while linear ARID1A expression remained unchanged (*p* > 0.05). As shown in Figure 2B, sh-circARID1A-1 exhibited better interference efficiency (*p* < 0.001), thus sh-circARID1A-1 was selected for subsequent experiments. qPCR results indicated that overexpression of circARID1A significantly reduced the mRNA levels of the adipogenesis marker genes adiponectin, CCAAT enhancer–binding protein A (CEBP/α), fatty acid–binding protein 4 (FABP4), and peroxisome proliferator-activated receptor γ (PPARγ) (*p* < 0.05), while knockdown of circARID1A had the opposite effect (*p* < 0.05) (Figure 2C). To further investigate its function, we examined the protein expression levels of these four adipogenesis marker genes, which were consistent with the mRNA results (*p* < 0.05) (Figure 2D,E). Additionally, Oil Red O staining showed that lipid droplet formation decreased with circARID1A overexpression and increased with circARID1A knockdown (Figure 2F). These results further indicate that circARID1A inhibits adipocyte differentiation.

### 2.3. circARID1A as a Molecular Sponge for miR-493-3p

**Figure 3:**
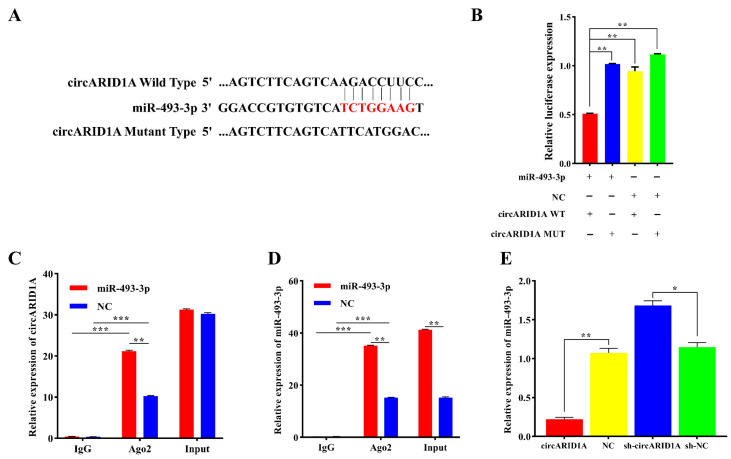
circARID1A acts as a molecular sponge for miR-493-3p. (**A**) Schematic diagrams of circARID1A-wt and circARID1A-mut luciferase reporters. (**B**) Luciferase activity in 293T cells co-transfected with circARID1A-wt or circARID1A-mut plasmids and miR-493-3p mimic or miR-493-3p NC for 48 h. (**C**,**D**) RIP assays in adipocytes using anti-AGO2 antibody or IgG, with qPCR measuring enrichment levels of circARID1A and miR-493-3p. (**E**) Expression levels of miR-493-3p after overexpression or knockdown of circARID1A. *: *p* < 0.05, **: *p* < 0.01, ***: *p* < 0.001.

Using RNAhybrid software (https://bibiserv.cebitec.uni-bielefeld.de/rnahybrid, accessed on 1 September 2024), we predicted binding sites between circARID1A and miR-493-3p (Figure 3A). To confirm the interaction between circARID1A and miR-493-3p, we constructed wild-type (circARID1A WT) and mutant (circARID1A MUT) plasmids containing the binding sites. These plasmids were co-transfected with miR-493-3p mimic (or miR-493-3p NC) into 293T cells. After 48 h of transfection, dual-luciferase reporter assays revealed that the luciferase activity of circARID1A WT was significantly inhibited (*p* < 0.01) (Figure 3B), whereas co-transfection of circARID1A MUT with miR-493-3p mimic did not affect luciferase activity (*p* > 0.05) (Figure 3B). These results indicate a target relationship between circARID1A and miR-493-3p. To further confirm this relationship, RIP assays were conducted using anti-AGO2 antibody and negative control IgG in adipocytes overexpressing miR-493-3p. qPCR analysis of the immunoprecipitated samples showed that the levels of circARID1A and miR-493-3p were significantly higher in AGO2-containing immunoprecipitates compared to IgG controls (*p* < 0.001) (Figure 3C,D). Additionally, in adipocytes transfected with miR-493-3p, the levels of circARID1A and miR-493-3p were significantly higher than those in controls (*p* < 0.01) (Figure 3C,D). Furthermore, interfering with circARID1A significantly increased miR-493-3p expression (*p* < 0.05), while overexpressing circARID1A significantly decreased miR-493-3p expression (*p* < 0.01) (Figure 3E). In summary, circARID1A likely acts as a molecular sponge for miR-493-3p.

### 2.4. miR-493-3p Regulates Adipocyte Differentiation

**Figure 4:**
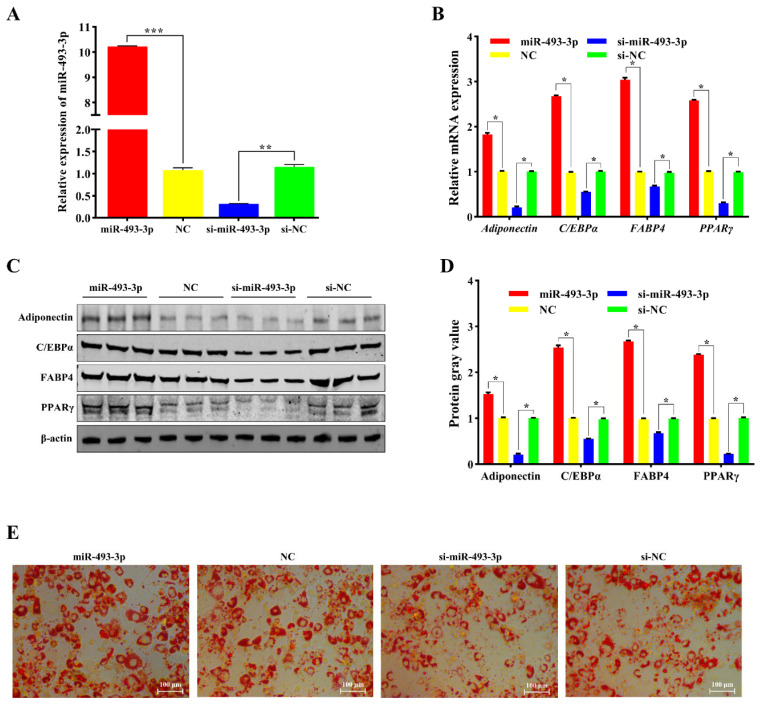
Effects of miR-493-3p on adipocyte differentiation. (**A**) Overexpression and inhibition of miR-493-3p. (**B**) mRNA expression levels of four adipocyte differentiation marker genes following overexpression and inhibition of miR-493-3p. (**C**,**D**) Protein expression levels of the four adipocyte differentiation marker genes after miR-493-3p overexpression and inhibition. (**E**) Oil Red O staining results of adipocytes after 6 days of differentiation. *: *p* < 0.05, **: *p* < 0.01, ***: *p* < 0.001.

To further investigate the role of miR-493-3p in adipocyte differentiation, we transfected the miR-493-3p mimic and miR-493-3p inhibitor into adipocytes (Figure 4A) and induced differentiation using differentiation medium after the cells were fully confluent. qPCR results showed that overexpression of miR-493-3p significantly increased mRNA levels of adiponectin, CEBP/α, FABP4, and PPARγ (*p* < 0.05) (Figure 4B), while inhibition of miR-493-3p resulted in significantly decreased levels (*p* < 0.05) (Figure 4B). Western blotting results were consistent with the qPCR findings (Figure 4C,D). After 6 days of differentiation, Oil Red O staining of the differentiated adipocytes revealed increased lipid droplets in cells overexpressing miR-493-3p, while those with miR-493-3p inhibition showed the opposite effect (Figure 4E). These results collectively indicate that miR-493-3p promotes adipocyte differentiation.

### 2.5. Target Relationship Between miR-493-3p and YTHDF2

**Figure 5:**
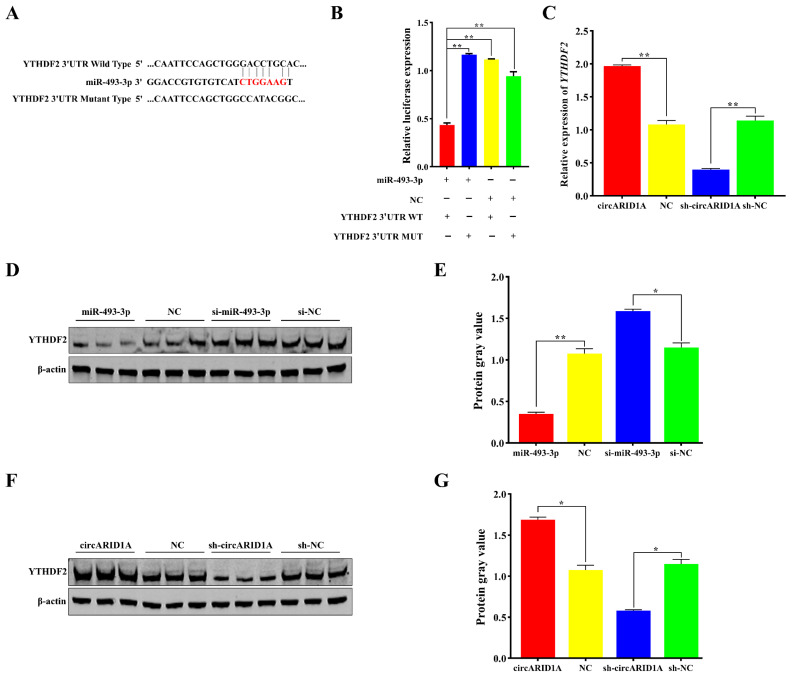
Target relationship between YTHDF2 and miR-493-3p, and regulation by circARID1A. (**A**) Schematic diagrams of YTHDF2 3′UTR WT and YTHDF2 3′UTR MUT luciferase reporters. (**B**) Luciferase activity measured 48 h after co-transfection of wild-type and mutant luciferase reporters with miR-493-3p mimic or miR-493-3p NC into 293T cells. (**C**) mRNA expression levels of YTHDF2 after overexpression or inhibition of circARID1A. (**D**,**E**) Protein expression levels of YTHDF2 after overexpression or inhibition of miR-493-3p. (**F**,**G**) Protein expression levels of YTHDF2 after overexpression or inhibition of circARID1A. *: *p* < 0.05, **: *p* < 0.01.

Using RNAhybrid software (https://bibiserv.cebitec.uni-bielefeld.de/rnahybrid, accessed on 1 September 2024), we predicted mRNAs with miR-493-3p binding sites and selected *YTHDF2*, a potential target gene related to lipid metabolism, for further experiments. We identified an miR-493-3p binding site in the 3′UTR of YTHDF2 (Figure 5A) and cloned both wild-type (YTHDF2 3′UTR WT) and mutant (YTHDF2 3′UTR MUT) sequences into the pmirGLO vector (Promega, Shanghai, China) to create dual-luciferase reporter constructs (Figure 5A). We then co-transfected miR-493-3p with these dual-luciferase reporters into 293T cells. Results showed that co-transfection of miR-493-3p with YTHDF2 3′UTR WT significantly reduced luciferase activity (*p* < 0.01) (Figure 5B), whereas co-transfection with YTHDF2 3′UTR MUT had no effect on luciferase activity (*p* > 0.05) (Figure 5B). Additionally, miR-493-3p significantly decreased the protein expression level of YTHDF2 (*p* < 0.05) (Figure 5D,E). In summary, miR-493-3p suppresses YTHDF2 expression by binding to its 3′UTR. Furthermore, we observed that overexpression of circARID1A significantly increased both mRNA and protein levels of YTHDF2 (*p* < 0.05) (Figure 5C,F,G), whereas interference with circARID1A had the opposite effect (*p* < 0.05) (Figure 5C,F,G). These results indicate that circARID1A indirectly regulates YTHDF2 expression through its interaction with miR-493-3p.

### 2.6. Regulation of Adipocyte Differentiation by YTHDF2

**Figure 6:**
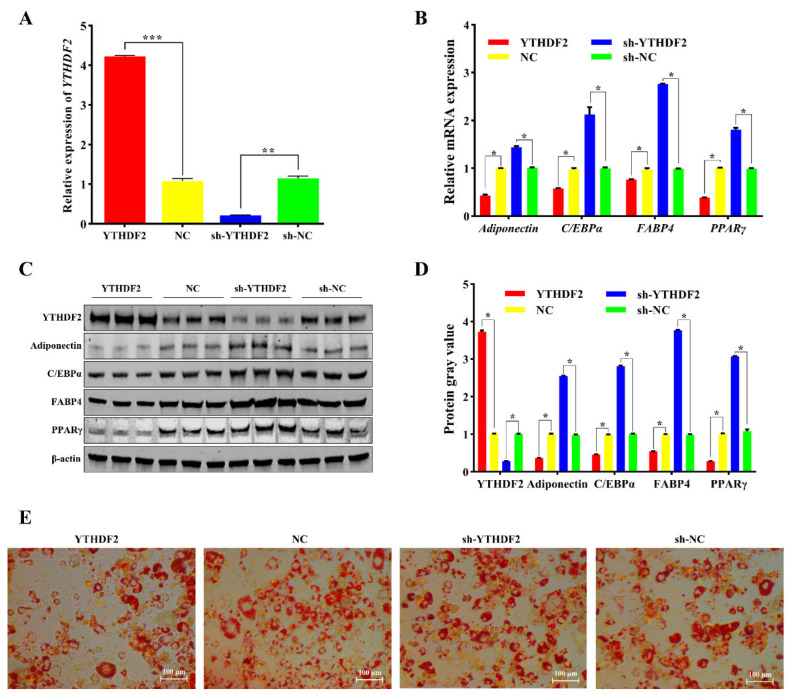
Effects of YTHDF2 on adipocyte differentiation. (**A**) Overexpression and knockdown of YTHDF2. (**B**) mRNA expression levels of four adipocyte differentiation marker genes following YTHDF2 overexpression and knockdown. (**C**,**D**): Protein expression levels of the four adipocyte differentiation marker genes after YTHDF2 overexpression and knockdown. (**E**) Oil Red O staining results of adipocytes after 6 days of differentiation. *: *p* < 0.05, **: *p* < 0.01, ***: *p* < 0.001.

Adipocytes were infected with lentivirus to achieve overexpression or knockdown of YTHDF2 (*p* < 0.01) (Figure 6A). Overexpression of YTHDF2 resulted in a significant decrease in the mRNA levels of adipocyte differentiation markers adiponectin, CEBP/α, FABP4, and PPARγ (*p* < 0.05) (Figure 6B), whereas knockdown of YTHDF2 had the opposite effect (*p* < 0.05) (Figure 6B). Western blot results were consistent with the qPCR findings (*p* < 0.05) (Figure 6C,D). Oil Red O staining showed that YTHDF2 overexpression led to fewer lipid droplets, while YTHDF2 knockdown resulted in more lipid droplets (Figure 6E). In summary, YTHDF2 inhibits adipocyte differentiation.

### 2.7. circARID1A/miR-493-3p/YTHDF2 Axis Regulates Adipocyte Differentiation

**Figure 7:**
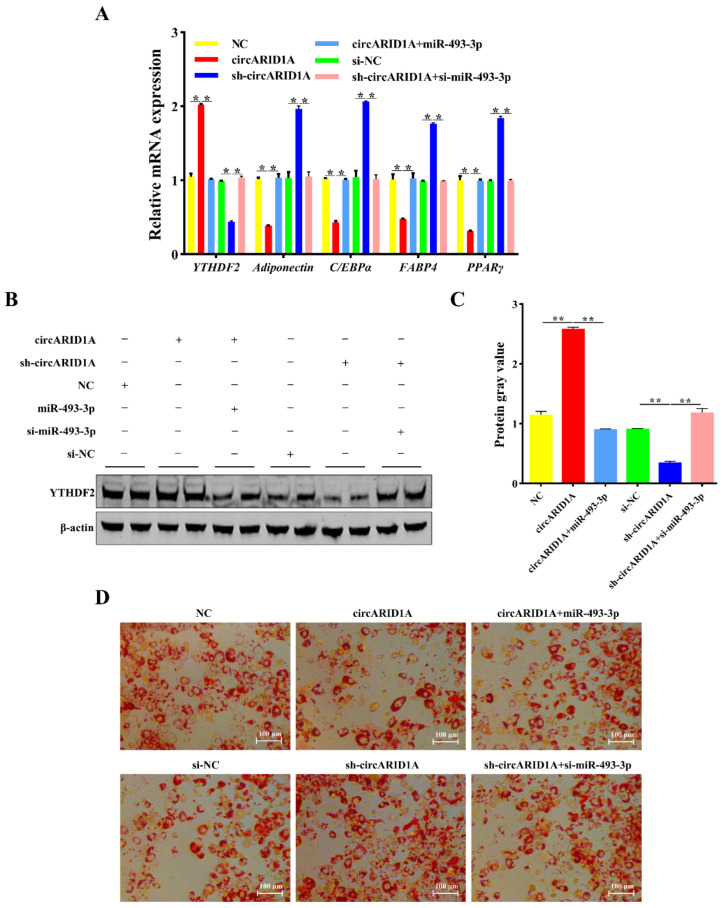
miR-493-3p Alleviates the regulation of YTHDF2 by circARID1A. (**A**) mRNA expression levels of YTHDF2 and adipocyte differentiation marker genes in the rescue experiment. (**B**,**C**) Protein expression levels of YTHDF2 in the rescue experiment. (**D**) Oil Red O staining results of adipocyte differentiation after 6 days in the rescue experiment. *: *p* < 0.05, **: *p* < 0.01.

To investigate whether circARID1A regulates adipocyte differentiation through the circARID1A/miR-493-3p/YTHDF2 axis, we performed rescue experiments by co-transfecting circARID1A and miR-493-3p into adipocytes. qPCR and Western blot analyses showed that upregulation of circARID1A significantly increased both mRNA and protein levels of YTHDF2 (*p* < 0.05) (Figure 7A–C), and these effects induced by circARID1A overexpression could be reversed by miR-493-3p overexpression (Figure 7A–C). Conversely, downregulation of circARID1A reduced YTHDF2 mRNA and protein levels (*p* < 0.05) (Figure 7A–C), and inhibition of miR-493-3p reversed these effects (Figure 7A–C). Additionally, the repressive effect of circARID1A on four adipocyte differentiation marker genes—adiponectin, C/EBPα, FABP4, and PPARγ—was diminished with miR-493-3p overexpression (Figure 7A). Oil Red O staining similarly showed that circARID1A overexpression reduced lipid droplet formation, which was counteracted by miR-493-3p (Figure 7D). Therefore, miR-493-3p reversed the inhibitory effect of circARID1A on adipocyte differentiation. In summary, circARID1A acts as a molecular sponge for miR-493-3p to regulate YTHDF2 expression, negatively modulating adipocyte differentiation in the tail fat cells of the Guangling Large-Tailed sheep.

## 3. Discussion

In recent years, the Guangling Large-Tailed sheep, known for its significant tail fat deposition, has become an important animal model for studying lipid accumulation [15]. The high tail fat content provides a unique perspective for understanding the mechanisms of adipocyte differentiation and lipid metabolism [16]. Previous studies indicate that tail fat development in this sheep breed primarily occurs around 6 months of age. Therefore, this study utilizes sequencing data from the tail adipose tissue of 6-month-old Guangling Large-Tailed sheep as the foundation for analysis, as fat accumulation at this stage provides a critical entry point for understanding the molecular mechanisms of adipocyte differentiation. Furthermore, the temporal variation in fat deposition may have profound effects on adipocyte differentiation and overall metabolism. Future research will thus focus on the characteristics of tail fat deposition across different age stages in the Guangling Large-Tailed sheep. Genomic and transcriptomic analyses help reveal key genes and signaling pathways involved in tail fat deposition [17,18]. This study, based on existing transcriptomic data, identified a circRNA specifically expressed in tail fat cells of the Guangling Large-Tailed sheep. As a novel type of non-coding RNA, circRNA plays a crucial role in regulating gene expression, protein translation, and interactions with other RNA molecules [19,20,21]. Its regulatory mechanisms in adipocyte differentiation have garnered increasing attention.

circRNAs are formed by the back-splicing of precursor mRNAs, resulting in a circular structure that is resistant to nuclease degradation, thus exhibiting high stability within cells [22]. In this study, we identified a circRNA specifically expressed in the tail fat cells of the Guangling Large-Tailed sheep, named circARID1A. Sanger sequencing confirmed that circARID1A is generated by back-splicing of exons 2 and 4 of the *ARID1A* gene. Similar to other circRNAs [23], circARID1A shows greater stability than linear RNAs under RNase R and ActD treatment due to its circular structure. Previous studies indicate that the functions of circRNAs are often related to their source genes [24]. ARID1A is involved in lipid metabolism by regulating PPARα expression [12]. Additionally, ARID1A affects the generation of free fatty acids and lipid droplets by regulating the PI3K-SREBP1 signaling pathway [25]. Therefore, investigating the role of circARID1A in lipid deposition and energy metabolism in adipocytes is of significant importance. Our experimental results suggest that circARID1A inhibits adipocyte differentiation, which aligns with the known functions of ARID1A in lipid metabolism. However, the specific mechanisms by which circARID1A, as a non-coding RNA, exerts its effects still require further investigation.

circRNAs exhibit diverse functions [26,27]. Among these, cytoplasmic circRNAs primarily act as “molecular sponges” [28], binding to specific miRNAs and inhibiting their expression. This prevents miRNAs from interacting with their target genes, thereby promoting the expression of miRNA target genes and regulating biological processes such as cell proliferation [29], differentiation [30], and metabolism [31]. Our study found that circARID1A is predominantly located in the cytoplasm, suggesting it may function as a “molecular sponge.” Bioinformatics analysis revealed binding sites between circARID1A and miR-493-3p (Figure 3A). Dual-luciferase reporter assays (Figure 3B) and anti-AGO2 RNA immunoprecipitation experiments (Figure 3C,D) confirmed the interaction between circARID1A and miR-493-3p. Previous research has shown that miR-493-3p is differentially expressed in subcutaneous fat tissues of Duolang sheep and Small Tail Han sheep, and regulates lipid metabolism by targeting AKT2 [32]. miR-493-3p modulates lipid metabolism by interacting with PDK4, inhibiting the pyruvate dehydrogenase complex, which catalyzes the conversion of pyruvate to acetyl-CoA, thereby reducing glucose utilization and enhancing fatty acid oxidation [33,34]. Additionally, miR-493-3p affects insulin synthesis and secretion by regulating IGF1R and its downstream effector MAPK1, thereby influencing lipid metabolism [35]. In summary, miR-493-3p participates in the comprehensive regulation of lipid metabolism through various mechanisms. Consistent with this, our study indicates that miR-493-3p can also promote adipocyte differentiation. However, the specific mechanisms by which miR-493-3p regulates adipocyte development remain unclear and warrant further investigation.

Recent studies have extensively reported that miRNAs regulate gene expression levels by targeting the 3′UTR of mRNAs [36,37,38]. Bioinformatics analyses revealed binding sites between the seed sequence of miR-493-3p and the 3′UTR of YTHDF2 mRNA. Our study relied on dual-luciferase reporter assays (Figure 5A,B) and quantitative PCR (Figure 5C,D,F) to validate the interaction between circARID1A and miR-493-3p. However, while the dual-luciferase assay provides preliminary confirmation of the targeting relationship, it does not fully reveal the specific regulatory mechanisms and dynamic changes involved in adipocyte differentiation. Considering the complex regulatory processes of adipocyte differentiation, future studies could incorporate higher-resolution molecular tools such as RNA pull-down and RIP-seq to further confirm the direct interaction between circARID1A and miR-493-3p and to elucidate the dynamic changes in this interaction across different differentiation stages. This would enhance the accuracy and biological relevance of the results. qPCR and Western blotting results showed that miR-493-3p downregulated YTHDF2 expression, indicating that miR-493-3p is a significant negative regulator of YTHDF2 in adipocytes. Furthermore, YTHDF2, as an m6A RNA methylation reader protein, selectively recognizes m6A modifications and mediates the degradation of m6A-containing mRNAs [39,40]. Specifically, YTHDF2 reduces the expression of CCNA2 and CDK2 by recognizing and degrading methylated mRNA, which promotes the cell cycle and inhibits adipogenesis [41]. Additionally, YTHDF2 recognizes and binds to the m6A sites of FAM134B, shortening mRNA lifespan and inhibiting protein expression, thereby participating in lipid deposition [42]. Similarly, YTHDF2 affects the mRNA stability and expression of tyrosine kinase 1 (JAK1) through an m6A-dependent mechanism, influencing the JAK1/STAT 5/C/EBPβ pathway and inhibiting adipogenic differentiation of bone marrow mesenchymal stem cells (BMSCs) [43]. We investigated the impact of YTHDF2 on adipocyte differentiation. Results from qPCR, Western blotting, and Oil Red O staining demonstrated that YTHDF2 inhibits adipocyte differentiation and lipid droplet accumulation. Additionally, we observed that circARID1A regulates the mRNA and protein levels of YTHDF2. We hypothesize that circARID1A may regulate YTHDF2 expression by competitively binding to miR-493-3p, thereby inhibiting adipocyte differentiation. To test this, we performed rescue experiments by co-transfecting circARID1A and miR-493-3p into adipocytes. Overexpression of circARID1A significantly increased YTHDF2 expression and inhibited adipocyte differentiation, while these effects were reversed by overexpressing miR-493-3p. In summary, circARID1A acts as a sponge for miR-493-3p, alleviating its suppression of the target gene YTHDF2, and thus inhibits adipocyte differentiation in sheep. In the next steps, proteomics analysis and more refined protein interaction assays can be employed to investigate whether circARID1A plays a role through additional pathways during adipocyte differentiation. This approach would further elucidate its multidimensional role in metabolic regulation.

Despite the progress made in this study, there are still some limitations. First, miR-493-3p and YTHDF2 represent only one of the targets and pathways through which circARID1A acts. The additional potential functions and regulatory pathways of circARID1A remain largely unexplored. This incompleteness could lead to a biased understanding of circARID1A’s role in adipocyte differentiation and lipid metabolism. Furthermore, this study primarily validated findings using in vitro cell models, lacking the support of in vivo experiments—a limitation that may impact the applicability of the results. In the cell model, the simplified experimental environment cannot fully replicate the complex in vivo microenvironment and intercellular interactions, which may prevent the results from fully reflecting circARID1A’s true functions in living organisms. This design limitation could lead to a partial understanding of circARID1A’s regulatory role in adipocyte differentiation and lipid metabolism. Additionally, factors such as treatment conditions and cell type selection in cell experiments may interfere with assessments of circARID1A’s regulatory effects. Therefore, conducting in vivo studies is essential to comprehensively understand the role of circARID1A in adipocyte differentiation and lipid metabolism. Future research will focus on in vivo studies, including gene knockout and overexpression experiments, as well as in vivo imaging. Through these studies, we aim to gain a more comprehensive and in-depth understanding of the specific mechanisms of circARID1A in adipocyte differentiation and lipid metabolism, providing new perspectives and methods for studying the molecular mechanisms of tail fat deposition in the Guangling Large-Tailed sheep.

## 4. Materials and Methods

### 4.1. Ethics Statement

In this study, all experimental procedures were conducted in strict accordance with the guidelines of the College of Animal Science and Veterinary Medicine at Shanxi Agricultural University. The experimental protocols were reviewed and approved by the institution’s Ethics Committee (Approval No.: SXAU-EAW-2022S.UV.010009), ensuring that animal pain and discomfort were minimized to the greatest extent possible. Samples of the Guangling Large-Tailed sheep were sourced from certified breeding facilities, and the entire sampling process was conducted under supervision, following the “3R” principles (replacement, reduction, and refinement) to minimize animal usage and optimize experimental conditions. Sampling procedures were performed on 3-day-old lambs as necessary for this study. Prior to sampling, all sheep underwent a comprehensive health assessment to confirm suitability for inclusion in this study. Stunning was performed using electric shock to reduce pain, followed by rapid exsanguination to achieve humane euthanasia. All procedures were carried out by professionally trained personnel to ensure compliance with standardized protocols and fulfillment of animal welfare requirements. Additionally, appropriate disinfection measures were implemented before and after sampling, and strict environmental controls were maintained to avoid unnecessary stress on the animals. All experimental instruments met sterility requirements to ensure the safety of both the animals and the experimental environment.

### 4.2. Cell Isolation and Culture

Guangling Large-Tailed sheep, provided by the sheep farm in Guangling County, Datong, Shanxi, were used as the experimental animals. Three 3-day-old male lambs were selected for this study due to the higher proportion of preadipocytes in their adipose tissue at this age, which enhances their differentiation potential and aligns with the experimental objectives. All lambs were born in December, corresponding with the reproductive seasonality of the Guangling Large-Tailed sheep, which predominantly produce winter lambs. This seasonal timing was chosen to ensure physiological consistency and environmental stability for the study subjects. The lambs were humanely euthanized via exsanguination following an electric shock. Tail fat tissues were collected, placed in PBS containing 1% penicillin-streptomycin (Gibco, Waltham, MA, USA), and refrigerated for transport to the laboratory. The collected fat tissues were rinsed in 75% ethanol, transferred to a dish with PBS, and cut into small pieces using ophthalmic scissors. The tissues were digested with 2% type II collagenase (Solarbio, Beijing, China) at 37 °C and 200 rpm for 30 min. Digestion was terminated with complete culture medium (10% fetal bovine serum (FBS; 04-001-1A, Biological Industries, Kibbutz Beit Haemek, Israel), 1% penicillin-streptomycin (10,000 U/mL penicillin and 10,000 μg/mL streptomycin; 15140122; Gibco, Waltham, MA, USA), and Dulbecco’s Modified Eagle Medium (DMEM; C11995500BT, Gibco, Waltham, MA, USA). The digestion mixture was centrifuged at 1000 rpm for 10 min at room temperature. The supernatant and floating fat were discarded, and the pellet was resuspended in complete culture medium. The suspension was filtered sequentially through 70 μm and 40 μm cell strainers (Solarbio, Beijing, China). The resulting cells were counted and plated in 10 cm^2^ culture dishes with 10 mL of complete culture medium, and incubated at 37 °C in a 5% CO_2_ incubator (Thermofisher, Waltham, MA, USA). After 6 h, the adherence of cells was checked, and the medium was replaced if cells had adhered. The medium was then replaced every 48 h. In this experiment, we used specific types of cell culture plasticware, including sterile, cell culture-treated polystyrene dishes (Corning, Corning, NY, USA). All dishes were sterilized by UV irradiation prior to the experiment to ensure aseptic conditions. Cells were cultured under specific conditions: 37 °C, 5% CO_2_, and 95% humidity in an incubator. The culture medium was replaced every 48 h to maintain sufficient nutrient availability. Throughout the culture process, all procedures were conducted in a sterile environment, and the medium was filtered before use to eliminate any potential contaminants that could impact experimental outcomes.

### 4.3. Vector Construction and Transfection

The full sequence of circARID1A was integrated into the pCD2.1-ciR vector (General Blol, Anhui, China), with a vector lacking the full-length circARID1A sequence serving as a control. This vector is capable of efficiently expressing the target gene in mammalian cells. The *GFP* gene was used to exclude the influence of the vector itself and verify the transfection efficiency. shRNA targeting circARID1A was synthesized by RiboBio (Guangzhou, China) (Table 1). The miR-493-3p mimic and inhibitor were synthesized by Sangon (Shanghai, China) (working concentration: 20 pmol/L). YTHDF2 3′UTR WT, YTHDF2 3′UTR MUT, circARID1A WT, and circARID1A MUT vectors were provided by Sangon (Table 2). Lipofectamine 3000 (Thermo Fisher Scientific, Waltham, MA, USA) was used as the transfection reagent. Successful transfected cell populations were selected using puromycin. All cloned products were confirmed through PCR validation, restriction enzyme analysis, and sequencing to ensure the successful construction of the vector.

### 4.4. Lentivirus Infection

Lentiviruses were synthesized by Hytyce (Suzhou, China). Lentiviral vector production was carried out using HEK293T cells as the host cells. The virus was produced using a three-plasmid lentiviral packaging system. After transfection, the cells were cultured in DMEM for 48 h, and the supernatant was collected. To ensure the quality of the virus, we concentrated the virus by ultracentrifugation (20,000× *g*, 4 °C, 2 h) and determined the viral titer using the TCID50 method. The day before transduction, a certain number of adipocytes were seeded into culture plates. When the cells reached 60% confluence, the virus solution with a multiplicity of infection (MOI) of 20 was added to the plates, ensuring that the medium covered all the cells. The plates were then incubated overnight at 37 °C in a 5% CO_2_ incubator. After 16 h of infection, the medium was replaced with fresh medium, and the cells continued to be cultured. The virus infection efficiency was evaluated by observing GFP protein fluorescence under a microscope. After 48 h of selection and amplification following transfection, cells were purified using antibiotic selection. The infection efficiency typically reached 80–90%, ensuring the reliability of downstream experiments.

### 4.5. Cell Differentiation and Oil Red O Staining

When the confluence of adipocytes reached 80–90%, the medium was changed to differentiation medium. This medium contained 89% DMEM, 1% penicillin-streptomycin, 0.5 mM 3-isobutyl-1-methylxanthine (IBMX; I8450, Solarbio, Beijing, China), 1 μM dexamethasone (ID0170, Solarbio, Beijing, China), and 10 μM insulin (I8040, Solarbio, Beijing, China). The medium was replaced every 48 h.

Before staining with Oil Red O, the cells were washed three times with PBS and fixed with 4% paraformaldehyde (BOSTER, Wuhan, China) at room temperature overnight. After fixation, the cells were gently washed twice with PBS, then rinsed with 60% isopropanol for 5 s. Oil Red O working solution (prepared by mixing Oil Red O solution with sterile water in a 3:2 ratio) was added to each well for 1 h of staining. The cells were then washed three times with sterile water and photographed under a microscope (Leica, Wetzlar, Germany). Oil Red O staining was performed on regions with consistent area and cell number. The area of Oil Red O staining in each image was calculated using ImageJ software (version 1.8.0). A threshold was set to distinguish the fat region from the background, and the percentage of the stained area was calculated. All data were obtained from three independent experiments.

### 4.6. RNase R Treatment

Cells were treated with 5 U/μg RNase R (Geneseed, Guangzhou, China) according to the reagent’s protocol, incubated at 37 °C for 30 min. The expression levels of circRNA and linear RNA were then assessed by qPCR.

### 4.7. Actinomycin D Assay

Cells were treated with 2 μg/mL Actinomycin D (MilliporeSigma, Burlington, MA, USA) and incubated for 0, 4, 8, 12, and 24 h. The stability of circRNA and linear RNA was assessed using qPCR.

### 4.8. RNA and Genomic DNA Extraction and Quantitative Real-Time PCR

Total RNA from tissues and cells was extracted using the Trizol-chloroform-isopropanol method. The PARIS Kit (Thermo Fisher Scientific, Waltham, MA, USA) was used to separate nuclear and cytoplasmic RNA. RNA quality was assessed with a NanoDrop 2000 spectrophotometer (Thermo Fisher Scientific, Waltham, MA, USA), and RNA integrity was checked using 1% agarose gel electrophoresis. Reverse transcription was performed with the PrimeScript RT Reagent Kit with gDNA Eraser (Takara, Otsu, Japan). qPCR was conducted using the TB Green Premix Ex Taq^®^TMII Kit (Takara, Otsu, Japan) and analyzed with the Bio-Rad CFX Connect system (Bio-Rad, Hercules, CA, USA). The primer sequences used in this study are listed in Table 3 and Table 4.

### 4.9. Western Blotting

Protease inhibitors (Solarbio, Beijing, China), phosphatase inhibitors (Solarbio, Beijing, China), and phenylmethylsulfonyl fluoride (PMSF) (Solarbio, Beijing, China) were added to the protein lysis buffer (Solarbio, Beijing, China). After incubating on ice for 30 min, the lysate was transferred to a 1.5 mL centrifuge tube, and centrifuged at 4 °C, 12,000× *g* for 5 min. The supernatant was used as the protein solution. Proteins were then separated by SDS-PAGE and transferred to a nitrocellulose membrane (NC membrane, Solarbio, Beijing, China). The membrane was washed three times with PBS, each time for 5 min, then blocked with 5% non-fat milk in TBST (Solarbio, Beijing, China) at room temperature for 1 h. After blocking, the membrane was washed three times with TBST, each time for 5 min, and incubated overnight at 4 °C with a diluted primary antibody. Following this, the membrane was washed with TBST and incubated with a secondary antibody (1:10,000, bs-0295G-AP, LI-COR, Lincoln, NE, USA) for 2 h. After washing, the membrane was scanned using an Odyssey Clx imaging system (LI-COR, Lincoln, NE, USA) and analyzed with Image Studio Version 5.2 (LI-COR, Lincoln, NE, USA). The primary antibodies used were PPARγ (1:2000, 16643-1-AP, Proteintech, Wuhan, China), C/EBPα (1:2000, 18311-1-AP, Proteintech, Wuhan, China), FABP4 (1:1000, 12802-1-AP, Proteintech, Wuhan, China), adiponectin (1:1000, bs-0471R, Bioss, Beijing, China), YTHDF2 (1:5000, 24744-1-AP, Proteintech, Wuhan, China), and β-actin (1:1000, BS-0061R, Bioss, Beijing, China).

### 4.10. Dual-Luciferase Reporter Assay

According to the Dual-Luciferase Reporter Assay Kit (Thermo Fisher Scientific, Waltham, MA, USA), 293T cells were seeded into 12-well plates. When the cell density reached 70%, the cells were co-transfected with reporter plasmids YTHDF2 3′UTR WT, YTHDF2 3′UTR MUT, circARID1A WT, and circARID1A MUT using Lipofectamine 3000. The cells were co-transfected with either miR-493-3p mimic or miR-493-3p NC. The experiment was divided into four groups, with six replicates per group. After 48 h of incubation at 37 °C with 5% CO_2_, luciferase activity was measured using the Dual-Luciferase Reporter Assay System (Promega, Shanghai, China) for statistical analysis.

### 4.11. RIP

Following the manufacturer’s instructions, RNA immunoprecipitation (RIP) was performed using an RNA Immunoprecipitation Kit (BersinBio, Guangzhou, China). Anti-AGO2 antibody (BOSTER, Wuhan, China) or negative control IgG was used to immunoprecipitate the RNA. The RNA was then extracted from the immunoprecipitate and analyzed by qPCR to measure the expression levels of miR-493-3p and circARID1A.

### 4.12. Target Interaction Prediction

Binding sites between circARID1A and miR-493-3p, as well as between miR-493-3p and YTHDF2, were predicted using RNAhybrid (https://bibiserv.cebitec.uni-bielefeld.de/rnahybrid, accessed on 1 September 2024).

### 4.13. Statistical Analysis

Statistical analysis was performed using GraphPad Prism software (version 9.5; GraphPad, San Diego, CA, USA). All experiments were conducted in triplicate or more, and data are presented as mean ± standard error of the mean (Mean ± SEM). Differences between the experimental and control groups were evaluated using Student’s *t*-test, while one-way ANOVA was used to compare differences among multiple groups. A *p*-value of <0.05 was considered statistically significant.

## 5. Conclusions

This study demonstrates that circARID1A acts as a molecular sponge for miR-493-3p, inhibiting the expression of the downstream gene *YTHDF2* and thereby suppressing the differentiation of tail adipocytes in the Guangling Large Tail sheep. These findings enhance our understanding of circRNA as a crucial regulatory factor in sheep fat development and provide a foundation for further exploring the metabolic mechanisms of sheep fat. This also offers a potential regulatory approach for optimizing meat quality and fat deposition in sheep. In the future, the circARID1A/miR-493-3p/YTHDF2 regulatory axis may be applied to manage livestock fat deposition through dietary adjustments or genetic selection. This approach could serve as a reference for enhancing sheep growth performance, increasing lean meat yield, and improving overall health, thereby advancing practical applications in the livestock industry.

## Figures and Tables

**Table 1:** shRNA sequences of circARID1A.

Name	Sequences (5′-3′)
sh-circARID1A-1	F: CTTCCAGCTTGCCTCCATC
	R: GATGGAGGCAAGCTGGAAG
sh-circARID1A-2	F: TGCCTCCATCCAGTCCAATG
	R: CATTGGACTGGATGGAGGCA

**Table 2:** shRNA sequences targeting YTHDF2 ^†^.

Names	Sequences (5′-3′)
sh-YTHDF2-1 forward	GATCCACCGGTAGATGCAATGTTTGGACAACCTTCAAGAGAGGTTGTCCAAACATTGCATCTTTTTTTGAATTCG
sh-YTHDF2-1 reverse	AATTCGAATTCAAAAAAAGATGCAATGTTTGGACAACCTCTCTTGAAGGTTGTCCAAACATTGCATCTACCGGTG
sh-YTHDF2-2 forward	GATCCGCGTGGATTCATCTAACTATATTCAAGAGATATAGTTAGATGAATCCACGCTTTTTTG
sh-YTHDF2-2 reverse	AATTCGAATTCAAAAAAGCCTAGAGAACAACGAGAATATCTCTTGAATATTCTCGTTGTTCTCTAGGCACCGGTG

^†^ Underscored sequences indicate enzyme sites.

**Table 3:** The qPCR primers for application of mRNAs.

Name of Primers	GenBank Accession Number	Sequences (5′-3′)
circARID1A forward		CCATATGGCGGGACTAACCC
circARID1A reverse		TGAGATAACTCTGCTGTGCAT
*PPARγ* forward	NM_001100921.1	ATCTTGACGGGAAAGACGAC
*PPARγ* reverse		AAACTGACACCCCTGGAAGAT
*FABP*4 forward	NM_001114667.1	AAACTGGGATGGGAAATCAACC
*FABP*4 reverse		TGCTCTCTCGTAAACTCTGGTAGC
*Adiponectin* forward	NM_001308565.1	ATCCCCGGGCTGTACTACTT
*Adiponectin* reverse		CTGGTCCACGTTCTGGTTCT
*C/EBPα* forward	NM_001308574.1	TCCGTGGACAAGAACAGCAA
*C/EBPα* reverse		TCATTGTCACTGGTCAGCTCC
*β-Actin* forward	NM_001009784.3	TGATGATATTGCTGCGCTCG
*β-Actin* reverse		GGGTCAGGATGCCTCTCTTG
miR-493-3p		UGAAGGUCUACUGUGUGCCAGG
*U6* forward	XR_003587591.1	CTCGCTTCGGCAGCACA
*U6* reverse		AACGCTTCACGAATTTGCGT
*YTHDF2* forward	XM_027965412.3	ACAGGCATCAGTAGGGCAAC
*YTHDF2* reverse		TTATGACCGAACCCACTGCC

**Table 4:** Sequences of divergent and convergent primers for circARID1A.

Name	Sequences (5′-3′)
circARID1A convergent	F: CCATATGGCGGGACTAACCC
	R: CCATGGTACTCTGCGCTCG
circARID1A divergent	F: CCATATGGCGGGACTAACCC
	R: TGAGATAACTCTGCTGTGCAT

## Data Availability

All data generated or analyzed during this study are included in this published article. The data that support the findings of this study are available from the corresponding author upon request.
